# The Patterns of Failure and Prognostic Impact of Tumor Location in Patients Undergoing Reirradiation for Glioblastoma

**DOI:** 10.7759/cureus.68820

**Published:** 2024-09-06

**Authors:** Alexis N Reinders, Matthew Koshy, Mark Korpics

**Affiliations:** 1 Department of Radiation Oncology, University of Illinois College of Medicine at Chicago, Chicago, USA; 2 Department of Radiation Oncology, University of Chicago, Chicago, USA

**Keywords:** leptomeningeal spread, recurrent glioblastoma, reirradiation, retrospective cohort study, tumor location, ventricular opening

## Abstract

Introduction

RTOG 1205 is the only randomized study to evaluate the safety and efficacy of reirradiation (reRT) in recurrent glioblastoma (GBM). While this study showed that reRT was safe and improves progression-free survival (PFS), an improved approach to reRT is still needed. In this study, we report on patterns of failure and outcomes in a cohort of patients with recurrent GBM who underwent reRT. We hypothesize that patients at high risk of leptomeningeal spread (LMS) are not good candidates for reRT due to the risk of treatment-related toxicity without clinical benefit.

Methods

In this retrospective study, patients with recurrent GBM who underwent reRT at a single institution from 2015-2023 were included. Sociodemographic, treatment, and outcomes data were collected via chart review. Time to progression was defined as the time from the start of reRT to progression per the Response Assessment in Neuro-Oncology (RANO) criteria. Overall survival (OS) was defined as the time from the start of reRT to death. PFS and OS were estimated using the Kaplan-Meier method.

Results

Thirteen patients with recurrent GBM who underwent reRT were identified. The median age at diagnosis was 58 years. Six patients (46.2%) had tumors that were O^6^-methylguanine-DNA methyltransferase (MGMT) methylated, four (30.8%) were MGMT unmethylated, and three (23.11%) had unknown MGMT status. Eight patients underwent repeat resection after recurrence and before reRT. Most patients (n=7) received 35 Gy in 10 fractions with concurrent bevacizumab, while other patients were treated with 25-40 Gy in 5-15 fractions with grade 1 or less acute toxicity. Three patients were treated with tumor-treating fields. The median follow-up was five months. Median PFS was three months [95% confidence interval (95% CI): one to four months] and median OS was five months (95% CI, 1-8 months) as compared to 7.1 months and 10.1 months, respectively, on RTOG 1205. Five patients developed LMS after reRT, one patient died before progression, and the remaining seven patients all developed progression within one centimeter of the recurrent tumor. Of the patients who developed LMS, all had tumors abutting the ventricles and three underwent resection 2-17 months before reRT.

Conclusion

Patterns of failure suggest a potential treatment selection approach for patients with recurrent GBM, in which patients at high risk of LMS (tumor abutting ventricles with or without recent surgery) should not undergo reRT, while patients at low risk of LMS are good candidates for reRT. Furthermore, reRT could be administered with reduced margins given that all non-LMS recurrences were within 1cm of the original tumor. Additional studies are needed to validate this approach.

## Introduction

Glioblastoma multiforme (GBM) is the most common primary malignant brain tumor in adults, with an incidence rate estimated to be between three and four per 100,000 people [1]. GBM accounts for 14.6% of all primary central nervous system (CNS) tumors and 48.3% of all malignant primary CNS tumors [2]. GBM more commonly affects men and all patients with GBM have a poor prognosis [1-3]. The median survival time and five-year survival rate have been estimated to be between 11 and 23 months and 7%, respectively [1-3].

Based on tumor location and patient comorbidities, the standard initial treatment for patients with GBM consists of maximum safe surgical resection, whether it be gross total (GTR) or subtotal (STR) resection, followed by concurrent temozolomide (TMZ) and external beam radiation therapy (EBRT) [4,5]. Unfortunately, despite multimodality therapy, local recurrence occurs in about 90% of patients within the first two years. Supportive care is an option; however, many patients decide to undergo further, aggressive treatments, including additional surgical resection(s), salvage systemic chemotherapy, reirradiation (reRT), or a combination of treatments [6,7]. Currently, there is no standard of care for recurrent GBM, and treatment must be initiated on an individual basis [6]. 

Numerous modalities and dosing regimens have been investigated via retrospective analyses; however, no phase III randomized control trials have been performed [8]. Modalities include EBRT, stereotactic radiosurgery, and brachytherapy. RTOG 1205, the only prospective, phase II randomized multi-institutional study to evaluate the safety and efficacy of reRT in recurrent GBM, demonstrated that patients treated with reRT (35 Gy in 10 fractions) and concurrent bevacizumab had prolonged progression-free survival (PFS) (3.8 vs. 7.1 months) when compared to bevacizumab alone. Unfortunately, results showed no significant difference in overall survival (OS) (9.7 vs. 10.1 months) [9].

Leptomeningeal spread (LMS), a complication of GBM, often indicates a very poor prognosis with a median OS of less than two months with supportive care only [10-13]. Risk factors for the development of LMS include young age (35-45 years old), male sex, prolonged survival after initial diagnosis, infratentorial and pineal tumors, ventricular opening during surgery, distance from the ventricles, and specific histologic and molecular markers [gains at the 1p36 locus, loss of glial fibrillary acidic protein (GFAP) expression, O6-methylguanine-DNA methyltransferase (MGMT) methylation, astrocytic phenotype, and high Ki67/Mib1 expression). Large tumor size, multiple surgeries, and anti-angiogenic therapies have also been suggested as potential risk factors [10-13]. Importantly, these factors have yet to be studied explicitly in the reRT population of GBM patients. OS in GBM patients receiving reRT is inversely correlated with age and tumor size and is directly associated with the time between initial RT and reRT, a higher Karnofsky Performance Scale (KPS), and reRT doses greater than 41.4 Gy [14,15]. We examine the patterns of failure and outcomes in a cohort of patients with recurrent GBM who underwent reRT at a single institution.

## Materials and methods

This retrospective cohort study analyzed patterns of failure in patients who received reRT for recurrent GBM at a single institution from 2015 to 2023. Institutional Review Board approval was obtained before the initiation of our study. The inclusion criteria were as follows: patients diagnosed with recurrent GBM, treated per standard of care, and receiving reRT. Patients who did not receive reRT as a part of their treatment regimen and patients who received reRT therapy at other institutions were excluded. Between 2015 and 2023, 147 patients who received radiotherapy for a malignant neoplasm of the brain at the University of Illinois Hospital and Health Sciences were identified. Among these, 66 patients were diagnosed with GBM, and 13 patients received reRT for GBM.

Sociodemographic, treatment, and outcome data were collected via retrospective chart review from January to April 2024. This included but was not limited to sex, age, race, ethnicity, performance status, molecular markers, type of initial and additional surgery, time between surgery and RT, dose of RT received, dose of reRT received, systemic therapies, time to initial progression, and treatment course toxicities. Time to progression was defined as the time from the start of reRT to progression per the Response Assessment in Neuro-Oncology (RANO) criteria. OS was defined as the time from the start of reRT to death. PFS and OS were estimated using the Kaplan-Meier method. Our study aim is to generate hypotheses for further research to improve treatment selection for patients with recurrent GBM.

## Results

A total of 13 patients with recurrent GBM who underwent reRT for progression were identified. Table 1 presents their sociodemographic and treatment data. The median age at diagnosis was 58 years and most patients (n=11, 84.6%) were male. Six patients (46.2%) were White, five (38.5%) were Black, and four (30.8%) were Hispanic. The most common presenting symptom was headache (n=5, 38.5%) followed by new-onset seizure(s) (n=4, 30.8%). Other symptoms included aphasia (n=2, 15.4%), weakness (n=2, 15.4%), dysarthria (n=1, 7.7%), and gait disturbance (n=1, 7.7%). Many patients (n=5, 38.5%) experienced more than one symptom before diagnosis. Initially, four patients (30.8%) had GTR, seven (53.8%) had STR, one (7.7%) had a stereotactic biopsy, and one's status (7.7%) was unknown.

**Table 1:** Cohort characteristics *Unknown due to surgery being performed at an outside institution. +Timeline was after initial or reirradiation treatment was completed until death CI: confidence interval; CNS: central nervous system; ECOG: Eastern Cooperative Oncology Group; IDH: isocitrate dehydrogenase; IQR: interquartile range; MGMT: O6-methylguanine-DNA methyltransferase; RT: radiotherapy

Characteristics	
Number of patients	13
Sex, n (%)	
Male	11 (84.6%)
Female	2 (15.4%)
Age at diagnosis, years, n (%)	
30-39	1 (7.7%)
40-49	3 (23.1%)
50-59	4 (30.8%)
60-69	4 (30.8%)
70-79	1 (7.7%)
Race, n (%)	
White	6 (46.2%)
Black or African American	5 (38.5%)
Other	2 (15.4%)
Ethnicity, n (%)	
Hispanic, Latino/a, or Spanish origin	4 (30.8%)
Not of Hispanic, Latino/a, or Spanish origin	9 (69.2%)
Performance status, n (%)	
ECOG 0	2 (15.4%)
ECOG 1	7 (53.8%)
ECOG 2	4 (30.8%)
MGMT methylation status, n (%)	
MGMT methylated	6 (46.2%)
MGMT unmethylated	4 (30.8%)
MGMT unknown	3 (23.1%)
IDH mutation status, n (%)	
IDH wildtype	10 (76.9%)
IDH mutant	1 (7.7%)
IDH unknown	2 (15.4%)
Initial radiation treatment, n (%)	
60 Gy in 30 fx	7 (53.8%)
54 Gy in 27 fx	3 (15.4%)
40.05 Gy in 15 fx	1 (7.7%)
35 Gy in 10 fx	1 (7.7%)
30 Gy in 10 fx	1 (7.7%)
Received temozolomide with initial RT, n (%)	
Yes	13 (100%)
No	0 (0.0%)
Weeks between initial surgery and the start of RT, n (%)	
01-Feb	1 (7.7%)
02-Jun	6 (46.2%)
06-Oct	5 (38.5%)
Unknown^*^	1 (7.7%)
Course one toxicity, n (%)	
No acute toxicity	7 (53.8%)
One or more acute grade 1 toxicities	6 (46.2%)
Grade 1 skin toxicity	5 (38.5%)
Grade 1 CNS toxicity	2 (15.4%)
Initial surgery, n (%)	
Gross total resection	4 (30.8%)
Subtotal resection	7 (53.8%)
Stereotactic biopsy only	1 (7.7%)
Unknown^*^	1 (7.7%)
Systemic therapies, n (%)	
Temozolomide only	1 (7.7%)
Temozolomide and bevacizumab only	7 (53.8%)
Temozolomide and bevacizumab plus evolizumab	1 (7.7%)
Temozolomide and bevacizumab plus lomustine only	2 (15.4%)
Temozolomide and bevacizumab plus lomustine and etoposide	1 (7.7%)
Temozolomide and bevacizumab plus lomustine and topotecan	1 (7.7%)
Progression-free survival after initial RT, months, median (IQR)	20.8 (6.5-60.1)
Overall survival after initial RT, months, median (IQR)^+^	38 (17-65)
Additional resections, n (%)	
Gross total resection	5 (38.5%)
Subtotal resection	3 (23.1%)
None	5 (38.5%)
Reirradiation dose, n (%)	
12 Gy in 1 fx	1 (7.7%)
25 Gy in 5 fx	2 (15.4%)
30 Gy in 10 fx	1 (7.7%)
35 Gy in 10 fx	7 (53.8%)
40 Gy in 15 fx	1 (7.7%)
Reirradiation course toxicity, n (%)	
No acute toxicity	12 (92.3%)
Grade 1 skin toxicity	1 (7.7%)
Progression-free survival after irradiation, months, median (95% CI)	3 (1-4)
Overall survival after irradiation, months, median (95% CI)^+^	5 (1-8)
Tumor-treating fields used, n (%)	
Yes	3 (23.1%)
No	11 (84.6%)
Time to last follow-up from the end of reirradiation, weeks, median (IQR)	22.5 (9-35.8)

Nine of the 13 patients had undergone surgery at our institution and thus we had access to the specifics contained within their operative reports. Ventricular opening was noted in a total of three operative reports. The most common initial tumor locations were the right temporal (n=3, 23.1%) and right frontal lobe (n=3, 23.1%) or both (n=1, 7.7%). The most common regimen for initial RT consisted of 60 Gy in 30 fractions (n=5, 38.5%) with concurrent chemotherapy; however, other patients received 30-54 Gy in 10-27 fractions. Eleven (84.6%) of the 13 patients received TMZ as part of their initial treatment. Seven patients (53.8%) did not experience acute toxicity from the first RT course while five (38.5%) did. The reported acute toxicities were grade one skin toxicity (n=4, 30.8%) and grade one CNS toxicity (n=2, 15.4%). Reported skin toxicities included skin hyperpigmentation (n=4, 30.8%) and alopecia (n=1, 7.7%) while reported CNS toxicities included mild headaches (n=1, 7.7%), ataxia (n=1, 7.7%), gait instability (n=1, 7.7%), and word-finding difficulties (n=1, 7.7%).

The median time from the end of initial RT treatment to recurrence was 30 months [interquartile range (IQR): 7-48 months]. Six patients (46.2%) had tumors that were MGMT methylated, four (30.8%) were MGMT unmethylated, and three (23.11%) had unknown MGMT methylation status. Ten patients (76.9%) had tumors that were isocitrate dehydrogenase (IDH) wild-type, one (7.7%) was IDH mutant, and the other two IDH mutation statuses were unknown. Eight patients underwent repeat resection after recurrence and before reRT. Of those, three underwent STR and five underwent GTR. One patient underwent a resection at another institution, and the operative report could not be obtained. Of the available operative reports, only one was notable for ventricular opening. Most patients (n=7) received 35 Gy in 10 fractions with concurrent bevacizumab, while others received 25-40 Gy in 5-15 fractions. Three patients were treated with tumor-treating fields; one out of them was reported to use TTF less than 18 hours per day and compliance information was not reported for the other two patients. The most common secondary tumor locations included the left parietal (15.4%), left frontal (15.4%), right temporal (15.4%), and right frontal (15.4%) lobes. In our cohort, the median tumor size in greatest diameter was 2.7 cm (IQR: 1.7-4.25 cm). Twelve (92.3%) patients did not experience acute toxicity after their second course of RT while one (7.7%) patient had grade one skin toxicity marked by hyperpigmentation, desquamation, alopecia, and hypohidrosis.

**Figure 1:**
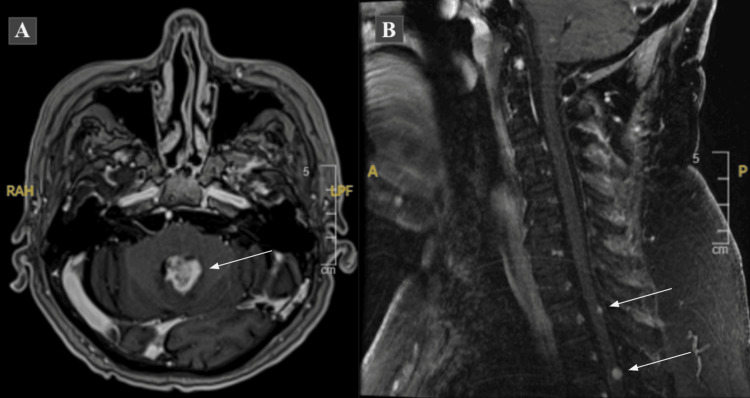
Leptomeningeal spread demonstrated on MRI Figure 1A: axial T1 post-contrast MRI of the brain demonstrating a heterogeneously enhancing mass occupying the fourth ventricle. Figure 1B: sagittal T2 FLAIR MRI of the cervical spine showing foci of nodular enhancement within the thoracic spine consistent with leptomeningeal spread FLAIR: fluid-attenuated inversion recovery; MRI: magnetic resonance imaging

The median follow-up was five months (IQR: 1.5-7.5 months), the median PFS was three months [95% confidence interval (95% CI): one to four months], and the median OS was five months (95% CI: one to eight months). Five (38.5%) patients developed LMS after reRT, one (7.7%) died before progression, and the remaining seven (53.8%) developed progression within 1 cm of the recurrent tumor (Figure 1). All who developed LMS had tumors abutting the ventricles; three (60%) were abutting a lateral ventricle and two (40%) were abutting the fourth ventricle. Most of the patients who developed LMS were males (80%), had MGMT methylated tumors (60%), and initially underwent STR (80%). Also, ventricular opening during initial surgical resection was seen in two (40%) who developed LMS. As for re-resection, there was one operative report that mentioned ventricular opening, in one of the patients with LMS. The median age of patients with LMS at the time of diagnosis was 68 years and the median recurrent tumor size in greatest diameter was 3.4 cm (IQR: 2.7-4.0 cm). Three patients who developed LMS underwent resection 2-17 months before reRT.

## Discussion

In our cohort, seven (53.8%) patients developed progression within 1 cm of the recurrent tumor; the other nine patients died before progression or developed LMS. The median age at the time of diagnosis was 68 years in those who developed LMS, older than the median age of 58 years in our entire cohort. The median recurrent tumor size in greatest diameter was 2.7 cm (IQR: 1.7-4.25 cm) while the median in our cohort with LMS was 3.4 (IQR: 2.7-4.0 cm) and the median in patients who did not develop LMS was 2.2 (IQR: 1.55-4.0 cm), indicating that tumor size is likely a risk factor for developing LMS. Our study suggests that ventricular opening during initial resection may be a significant risk factor, as two (40%) of the five of our patients who developed LMS had ventricular opening during their initial surgery. All five (100%) who developed LMS after reRT had tumors that abutted the ventricle(s). Our results suggest that distance from the ventricles is a significant risk factor for developing LMS, especially in the setting of reRT. Hence, patients with tumors abutting the ventricles should either not undergo reRT or undergo shorter, palliative courses of RT, as the risk of LMS outweighs the benefit of reRT and bevacizumab. However, more studies are needed to confirm this approach.

Tsien et al. demonstrated that recurrent GBM patients who received reRT plus bevacizumab vs. bevacizumab alone had prolonged PFS (7.1 vs. 3.8 months) but there was no significant difference in OS (10.1 vs. 9.7 months). Our median PFS and OS were three months and five months, as compared to 7.1 months and 10.1 months, respectively, reported in RTOG 1205. Notably, our PFS and OS were more comparable to the patients treated with bevacizumab only in RTOG 1205. Significantly, factors such as target volume, time between initial RT and reRT, initial tumor location, and distance from the third ventricle were not reported by RTOG 1205 [9]. However, these factors may have significantly affected the risk for LMS and thus the OS in certain patients [9,11,12,14,15]. Our study provides new insights by demonstrating that improved treatment selection may be advantageous for patients with recurrent GBM and those at high risk of LMS may benefit from alternative treatment plans instead of the RTOG 1205 regimen.

Larger clinical target volumes (CTVs) and dose escalation have been hypothesized to benefit patients who receive RT/reRT. However, in agreement with our findings, the location of GBM recurrence is most commonly in-field; therefore, larger CTVs are unlikely to lower the risk of recurrence [16-18]. Some studies suggest that reducing the CTV decreases the number of in-field recurrences and increases multifocal recurrence, which may be associated with decreased OS. Overall, larger CTVs would likely lead to decreased quality of life given the larger irradiated volume without significantly reducing the risk of recurrence [18]. As for dose escalation with initial RT, studies have shown mixed results [19,20].

Preliminary results from the ongoing trial (NRG Oncology BN001), which compared dose-intensified (DI) RT versus standard-dose (SD) RT with TMZ, demonstrated that the difference in OS between DI RT and SD RT did not meet the significance threshold for phase III trial consideration [21]. Since there is no agreed-upon standard dose for reRT, studies that investigate dose escalation in reRT are limited. A phase I dose escalation study of hypofractionated stereotactic reRT therapy with bevacizumab in 13 patients with anaplastic and recurrent GBM indicated that 11 Gy in three fractions is well tolerated and may lead to increased OS, although further studies are warranted [22]. Furthermore, a retrospective analysis of 52 reRT patients suggested the same; however, the median biological equivalent dose of 10 Gy (BED10) of reRT was 53.1 Gy [23]. In conclusion, more studies are needed to assess whether or not increasing the CTV size or dose of reRT prolongs PFS or OS.

While some studies suggest that surgical opening of and distance from the ventricles are risk factors for developing LMS, other studies refute this notion [12,24,25]. In a retrospective study, 11 patients with LMS after being treated with chemotherapy, RT, and surgical intervention for malignant gliomas were investigated for risk factors. All patients were found to have tumors that had a connection with either the pial or ventricular space and had a history of surgical opening of the ventricle [24]. In contrast, a retrospective analysis of 51 patients with high-grade supratentorial gliomas at the University of Washington showed that both surgical ventricular entry and the distance from the ventricle were not significantly associated with LMS [25]. Also, a retrospective analysis of 36 patients performed at the M.D. Anderson compared patient characteristics between groups of GBM patients who did and did not develop LMS. Characteristics included sex, KPS, location, extent of resection, number of surgeries, communicating with the ventricle at diagnosis, and ventricle opened on initial resection; none were shown to predict LMS. However, distance from the ventricle was found to be statistically significantly associated with a shorter time to LMS development from the initial diagnosis of GBM [12]. More studies are needed to determine if either surgical opening or distance from the ventricles are risk factors for LMS.

Previous research suggests that male sex and young age are risk factors for developing LMS in GBM patients [13,26]. Our study further suggests that male sex is a demographic risk factor for developing LMS; however, our observation differs from previous research that suggests that young age is a risk factor. Additionally, studies have suggested that large tumor sizes may be a risk factor for LMS, although the evidence is conflicting [12,13]. Other theorized causes of LMS include intrinsic factors, such as molecular differences between individual patient tumors which are thought to increase susceptibility. For instance, our study suggests patients (n=3, 60%) with MGMT methylated tumors are at greater risk of LMS. While studies have supported this notion, other studies have further suggested other molecular markers, such as gains at the 1p36 locus, high Ki67/Mib1 expression, and loss of GFAP expression [10-13,27].

The Internet of Things (IoT) is a novel concept that may offer new opportunities for managing GBM patients undergoing radiation therapy by connecting physical devices to the internet for streamlined data sharing and patient monitoring. For example, augmented reality could be used to virtually simulate the entire radiotherapy session and aid in patient positioning in real-time, thereby enhancing both the pre-planning simulations and treatment sessions [28,29].

Strengths and limitations

Strengths of our study include the recent time frame of data collection (2015-2023) and the high proportion of underrepresented minorities included in our study population, which are often not well represented in other studies. Limitations include the small sample size (n=13), which makes our results susceptible to both type I and II errors. Another limitation is the use of a retrospective study model, which is always at greater risk of misclassification, selection, or recall bias. Due to the nature of chart review, another limitation may involve missing or unreported data. Our data also represents patients who were treated at a single institution, which is also considered a limitation when compared to studies that use multi-institutional data.

## Conclusions

Our study involving patients with recurrent GBM treated with reRT at a single institution demonstrated numerically worse PFS and OS as compared to RTOG 1205. Our analysis of the patterns of failure suggests that a new treatment selection approach is possible for patients with recurrent GBM. More specifically, patients at high risk of LMS, such as those with tumors abutting the ventricles, should not undergo reRT because they will be exposed to RT toxicity without any PFS benefit. In contrast, patients at low risk of LMS are good candidates for reRT with reduced margins. Larger, multi-institutional studies are needed to validate this approach and better estimate treatment outcomes.
